# Surviving Without Nerves: Intracardiac Denervation as an Underrecognized Feature of Cardiac Allograft Vasculopathy and Chronic Graft Dysfunction: A Morphological Case Report 11 Years After Transplantation

**DOI:** 10.3390/diagnostics16162497

**Published:** 2026-08-07

**Authors:** Igor Makarov, Victoria Smolnikova, Anna Starshinova, Dmitry Kudlay, Maria Simonenko, Petr Fedotov, Lubov Mitrofanova

**Affiliations:** 1 Almazov National Medical Research Centre, 2, Akkuratov Str., 197341 St. Petersburg, Russia; smolnikovaviktoriaa@yandex.ru (V.S.); simonenko_ma@almazovcentre.ru (M.S.);; 2 Department of Mathematics and Computer Science, St-Petersburg State University, 199034 St. Petersburg, Russia; 3 Department of Pharmacology, Institute of Pharmacy, Sechenov University, 119002 Moscow, Russia; d624254@gmail.com; 4 NRC Institute of Immunology FMBA of Russia, 115522 Moscow, Russia; 5 Faculty of Bioengineering and Bioinformatics, Lomonosov Moscow State University, 119991 Moscow, Russia

**Keywords:** chronic rejection, heart transplantation, intracardiac innervation, denervation, cardiac allograft vasculopathy, neuroinflammation, immunohistochemistry, quantitative morphometric analysis

## Abstract

**Background/Objectives:** Orthotopic heart transplantation results in complete surgical denervation of the donor heart. Although partial functional reinnervation has been reported in some recipients, the histopathological features of intracardiac innervation in long-term failing cardiac allografts remain poorly characterized. We present a unique case of a cardiac allograft explanted 11 years after transplantation that enabled detailed histological and digital assessment of neural remodeling. **Case Description**: An explanted cardiac allograft obtained after retransplantation for chronic graft dysfunction was examined using whole-heart histopathological reconstruction and immunohistochemistry (CD3, CD68, C3, C4d, HLA-DR, NLRP3, VEGF, and UCHL-1). Digital quantitative analysis of UCHL-1-positive nerve fibers, including spatial density mapping and comparison with a reference non-diseased myocardium, demonstrated profound and heterogeneous loss of intracardiac innervation. Marked denervation was observed not only within fibrotic and infarcted regions but also in the morphologically preserved myocardium. Neural injury was associated with perineural and endoneurial inflammatory infiltrates, complement deposition, HLA-DR expression within nerve fibers and reduced perivascular and epicardial adipose tissue innervation. Distinct regional patterns of denervation and neural remodeling were also identified in the aortic segments of the transplantation complex. **Conclusions**: This case demonstrates that chronic cardiac allograft dysfunction may involve extensive injury of the intracardiac nervous system extending beyond areas of ischemic myocardial damage. The findings suggest that immune-mediated inflammation, complement activation, and chronic ischemia contribute to progressive neural remodeling in the transplanted heart. Histopathological evaluation of intracardiac innervation may provide additional insight into mechanisms of long-term graft failure and represents an underrecognized component of cardiac allograft pathology.

## 1. Introduction

Heart transplantation remains the gold standard treatment for end-stage heart failure refractory to medical therapy. Nevertheless, surgical denervation of the donor heart inevitably results in the loss of its autonomic innervation [1]. The absence of sympathetic and parasympathetic regulation substantially alters the physiology of the transplanted heart and the clinical phenotype of the recipient, manifesting as an elevated resting heart rate, silent ischemia, reduced exercise tolerance, and impaired adaptation of cardiac output to the metabolic demands of the organism [2,3]. In this context, the possibility and extent of reinnervation of the transplanted heart are of particular clinical importance, since restoration of neural regulation may potentially improve functional reserve and quality of life in transplant recipients [4].

For clarity, several related terms are used throughout this manuscript with distinct meanings. Surgical denervation refers to the immediate interruption of autonomic nerve fibers occurring during heart transplantation. Reinnervation denotes subsequent regrowth or restoration of neural connections after transplantation. Neural injury describes inflammatory or immune-mediated structural damage affecting existing nerve fibers, whereas secondary denervation refers to the loss of previously existing neural structures as a consequence of chronic graft pathology. Finally, neural remodeling is used as a broader term encompassing both degenerative and regenerative structural changes within the intracardiac nervous system.

Over the past three decades, a substantial body of evidence has accumulated indicating the possibility of partial reinnervation of the cardiac allograft. Various approaches have been employed to assess this process, including functional imaging using positron emission tomography (PET) and single-photon emission computed tomography (SPECT), pharmacological testing, and morphological examination of endomyocardial biopsy specimens.

Despite this methodological diversity, the available findings remain largely contradictory. On the one hand, several lines of evidence support the occurrence of partial reinnervation. PET studies using ^11^C-hydroxyephedrine (^11^C-HED) have demonstrated progressive increases in myocardial radiotracer uptake with longer post-transplant follow-up, which has been interpreted as evidence of restoration of functionally active sympathetic nerve terminals [5,6]. Furthermore, some recipients exhibit improved functional performance during exercise testing, including significantly higher peak heart rate, greater chronotropic reserve, and increased maximal oxygen consumption compared with patients without evidence of reinnervation [4,7]. Morphological studies have also demonstrated the presence of nerve fibers within myocardial biopsy specimens in a substantial proportion of patients (up to 65% in certain case series), as confirmed by immunohistochemical detection of neuronal markers [8]. Additional neurochemical evidence, including assessment of the coronary venous gradient of dihydroxyphenylglycol (DOPEG), a presynaptic metabolite of noradrenaline, further suggests the presence of functionally active sympathetic nerve endings within the transplanted heart [9].

On the other hand, substantial evidence calls into question both the completeness of reinnervation and its clinical relevance. First, even when present, reinnervation appears to be fragmentary and regionally restricted: in most imaging studies, radiotracer uptake is observed predominantly in the anterior and septal walls of the left ventricle, whereas the posterior and lateral segments remain denervated [10,11]. Second, marked interindividual variability has been reported, with some patients demonstrating no evidence of reinnervation even 10–13 years after transplantation [1]. Third, quantitative indices of sympathetic ligand uptake within reinnervated regions remain considerably lower than normal physiological values, raising doubts regarding the functional adequacy of the restored innervation [12,13]. Fourth, discrepancies have been identified between different methods of assessment, for example, preserved myocardial contractile responses to adrenergic stimulation despite minimal or absent uptake of ^123^I-metaiodobenzylguanidine (^123^I-MIBG), which may reflect the direct action of circulating catecholamines on cardiomyocytes independently of neural fibers. Additional factors potentially influencing reinnervation include recipient age at the time of transplantation, characteristics of immunosuppressive therapy, and the development of cardiac allograft vasculopathy [14,15].

Particular attention should be paid to the morphological assessment of nerve fibers in endomyocardial biopsy specimens. This approach remains controversial, since endomyocardial biopsy primarily permits evaluation of subendocardial neural structures, whose functional role is considerably less well understood than that of the sinoatrial and atrioventricular nodal regions. Moreover, the mere identification of a nerve fiber cannot be regarded as proof of its functional activity. Likewise, the presence of neural structures does not unequivocally confirm reinnervation, as these fibers may represent residual donor-derived neural elements rather than newly established connections with the recipient’s extracardiac nervous system. Another major limitation is the absence of comparative data from endomyocardial biopsies obtained outside the transplantation setting, particularly given that nerve fibers are inconsistently and irregularly visualized even in cohorts of non-transplanted patients.

To date, most studies of cardiac reinnervation after transplantation have focused on demonstrating the presence or absence of sympathetic reinnervation using functional imaging, physiological testing, or limited endomyocardial biopsy specimens. In contrast, comprehensive pathological assessment of the intracardiac nervous system in explanted cardiac allografts with advanced chronic rejection has not been reported. The present study combines whole-heart histological reconstruction, quantitative nerve density mapping, immunohistochemical assessment of neural injury, and evaluation of the donor–recipient aortic transition, providing an integrated view of neural remodeling beyond the scope of previous imaging- or biopsy-based studies.

Thus, despite more than three decades of investigation, the issue of cardiac allograft reinnervation remains unresolved. The contradictory findings obtained using different methodologies, the fragmentary and regionally heterogeneous nature of reinnervation, and the uncertainty regarding its clinical significance underscore the need for further comprehensive studies integrating functional imaging, morphological analysis, and clinical outcome assessment. The aim of the present study was to investigate neural fibers within a cardiac allograft under conditions of chronic rejection.

## 2. Case Presentation

**Figure 1:**
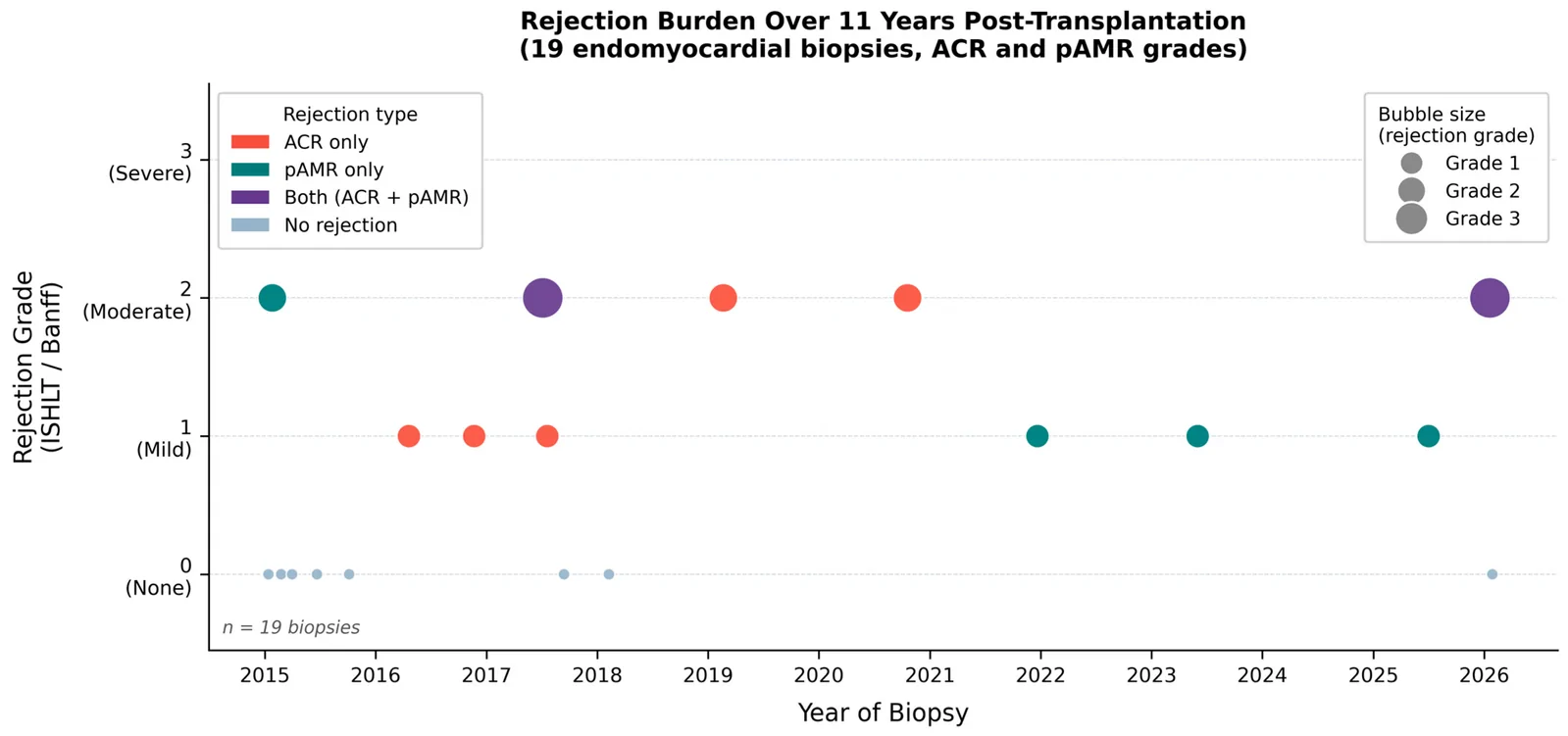
Burden of allograft rejection over an 11-year follow-up period.

A 30-year-old man with end-stage primary dilated cardiomyopathy underwent orthotopic heart transplantation using the bicaval technique. During the subsequent 11-year follow-up, graft surveillance was performed by serial endomyocardial biopsies. A total of 19 biopsies were available for review. Throughout most of the observation period, biopsies demonstrated either no evidence of rejection or mild acute cellular rejection (ACR grade 1R), with intermittent episodes of moderate cellular rejection (grade 2R). Beginning in December 2021, three consecutive biopsies demonstrated isolated pathological antibody-mediated rejection (pAMR1(H+)), indicating increasing humoral immune activity. Subsequently, two episodes of combined moderate acute cellular rejection (grade 2R) and antibody-mediated rejection (pAMR2) were documented (July 2017 and January 2026), suggesting progression toward chronic immune-mediated graft injury (Figure 1).

Despite immunosuppressive therapy, the patient gradually developed chronic cardiac allograft dysfunction complicated by acute myocardial infarction, ultimately requiring retransplantation 11 years, 2 months, and 13 days after the initial transplantation. The patient died 16 days after the second transplantation because of disseminated intravascular coagulation syndrome associated with severe infectious complications.

**Figure 2:**
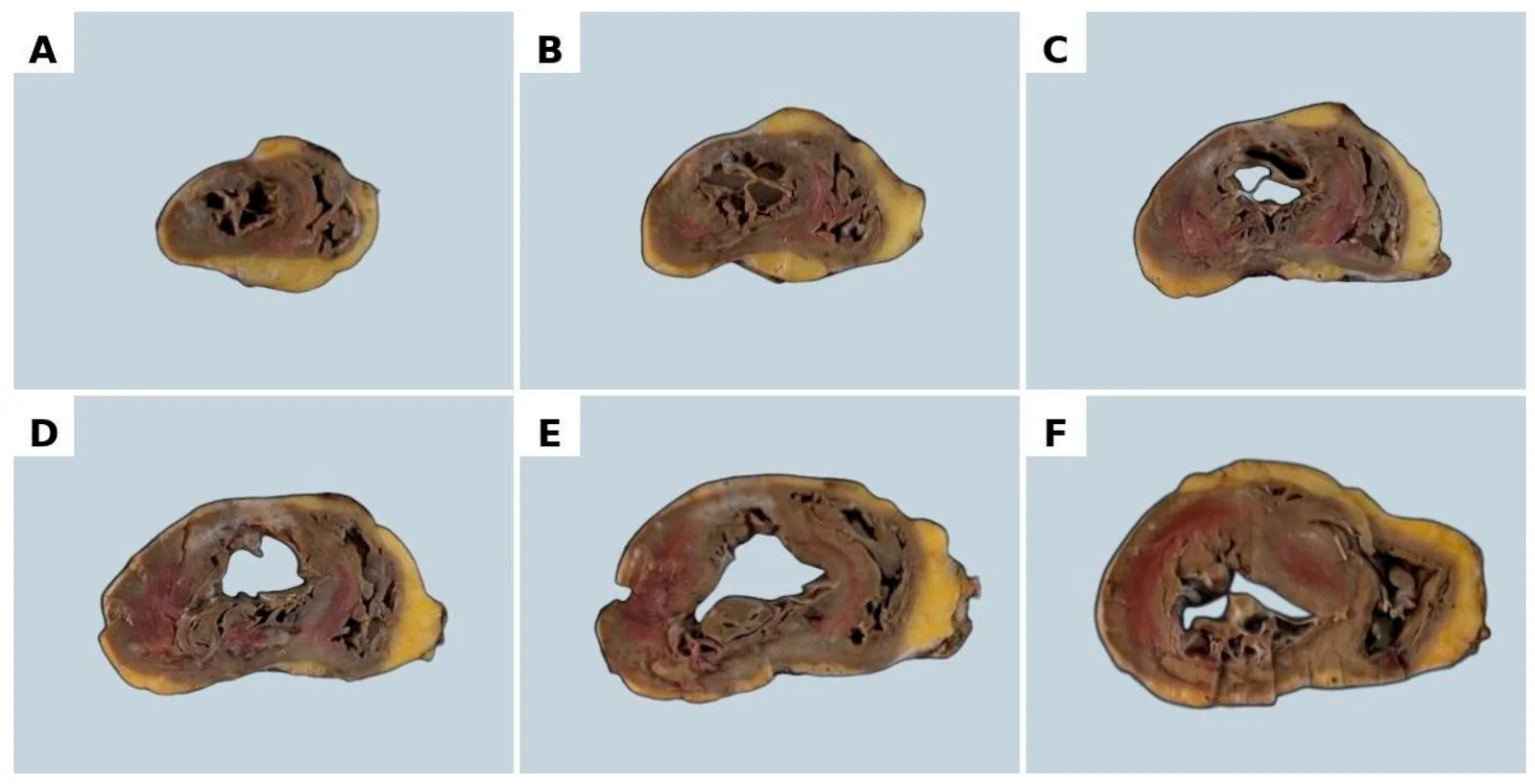
Gross appearance of the heart: transverse myocardial sections from the apex to the base (**A**–**F**). Scar formation involving the anterior wall and an area of necrosis within the posterior wall extending into the interventricular septum.

**Figure 3:**
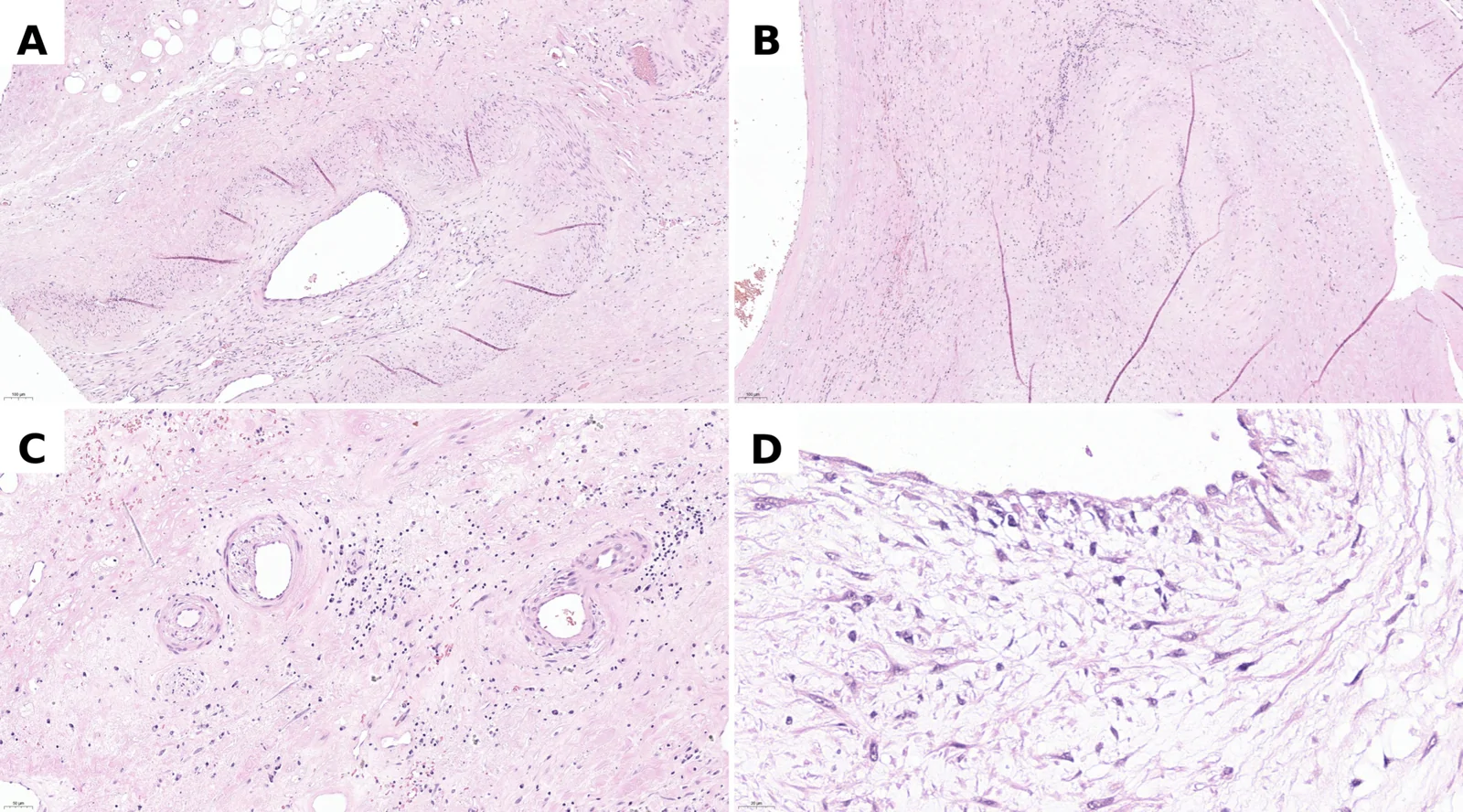
Histological vascular alterations, hematoxylin and eosin staining. (**A**) Transverse section of a diagonal artery at the mid-ventricular level of the left ventricle demonstrating marked neointimal proliferation and perivascular fibrosis, ×100; (**B**) section of the left anterior descending artery at the same level showing complete luminal obliteration caused by sclerosis and hyalinosis of the neointima with paravascular fibrosis, ×100; (**C**) multiple thick-walled coronary arterial branches with intimal proliferation embedded within fibrotic tissue, ×200; (**D**) neointimal proliferation of polymorphic cells in a diagonal artery, ×630.

Morphological examination of the first transplanted heart confirmed severe cardiac allograft vasculopathy, post-infarction changes involving the anterior wall, and an extensive myocardial infarction of 2–3 weeks’ duration affecting the posterior and lateral walls with involvement of the interventricular septum. For the purposes of analysis, a left ventricular myocardial ring at the mid-ventricular level was selected, in which the area of scar tissue did not exceed 10% and the area of recent infarction did not exceed 20% (Figure 2 and Figure 3).

Morphological examination revealed a combination of severe post-transplant vasculopathy and profound injury to myocardial and epicardial neural structures developing in the setting of chronic immune-mediated rejection of the allograft.

**Figure 4:**
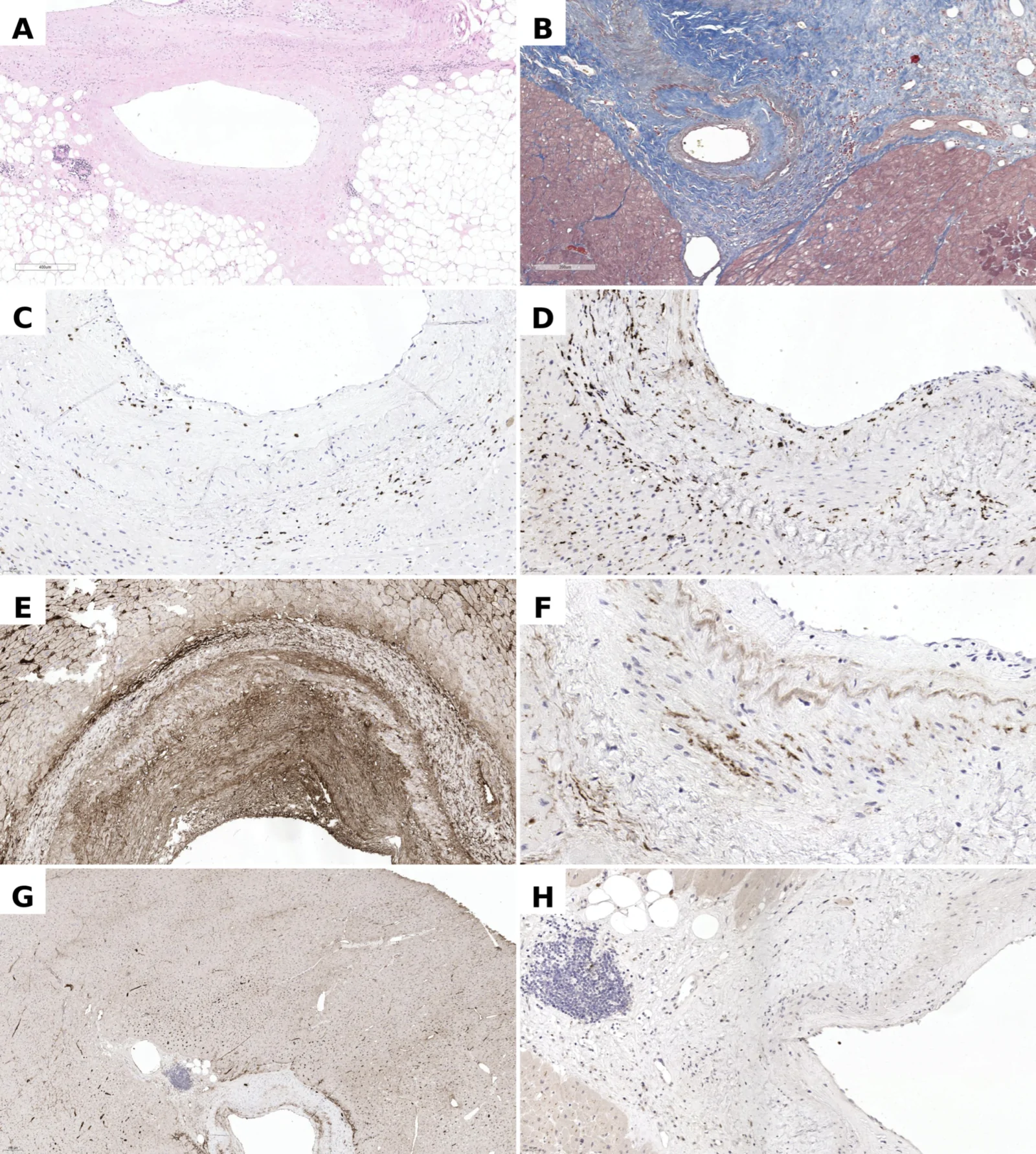
Morphological examination of vessels in post-transplant vasculopathy. (**A**) Marked perivascular fibrosis with scarring at the sites of prior nerve fibers at the periphery of an epicardial artery, hematoxylin and eosin staining, ×50; (**B**) perivascular fibrosis, intimal fibrosis, and absence of nerve fibers and ganglia at the arterial periphery, Masson’s trichrome staining, ×100; (**C**) CD3 immunostaining demonstrating CD3+ cells within the vascular wall and perivascular region, ×200; (**D**) CD68 immunostaining showing infiltration by CD68+ macrophages predominantly in the adventitia and intima of the vessel with few macrophages in the media, ×200; (**E**) deposition of complement component C3 under the endothelium, along the medial fibers and in the adventitia, ×200; (**F**) C4d deposition within the vascular wall and in macrophages, ×400; (**G**) gradient expression of VEGF with a peak at the vascular periphery and gradual attenuation towards the periphery, ×50; (**H**) NLRP3 expression in the cytoplasm of a small number of inflammatory cells both within and around the vessel, ×200.

Cardiac allograft vasculopathy was characterized by diffuse neointimal proliferation in large coronary and intramyocardial arteries, loss of elastic laminae, fibrosis of the muscular layer, and marked perivascular fibrosis. At the periphery of the vessels, dense lymphoplasmacytic infiltrates were observed, with the formation of lymphoid aggregates lacking germinal centers. Immunohistochemically, intense expression of complement components C3 and C4d, as well as Human Leukocyte Antigen-DR isotype (HLA-DR), was detected in the intima and vascular wall, indicating a significant contribution of humoral rejection mechanisms. The inflammatory infiltrate consisted predominantly of CD3+ T lymphocytes and CD68+ macrophages. In addition, Target of Methylation-induced Silencing 1 (TMS1)- and Nucleotide-binding oligomerization domain, Leucine-rich Repeat and Pyrin domain containing (NLRP3)-positive cells were identified, suggesting activation of the inflammasome cascade and pyroptotic pathways. Marked neoangiogenesis was observed at the periphery of affected vessels, with a characteristic gradient of Vascular Endothelial Growth Factor (VEGF) expression: highest in proximity to the vascular wall and gradually decreasing towards the myocardial interstitium (Figure 4).

**Figure 5:**
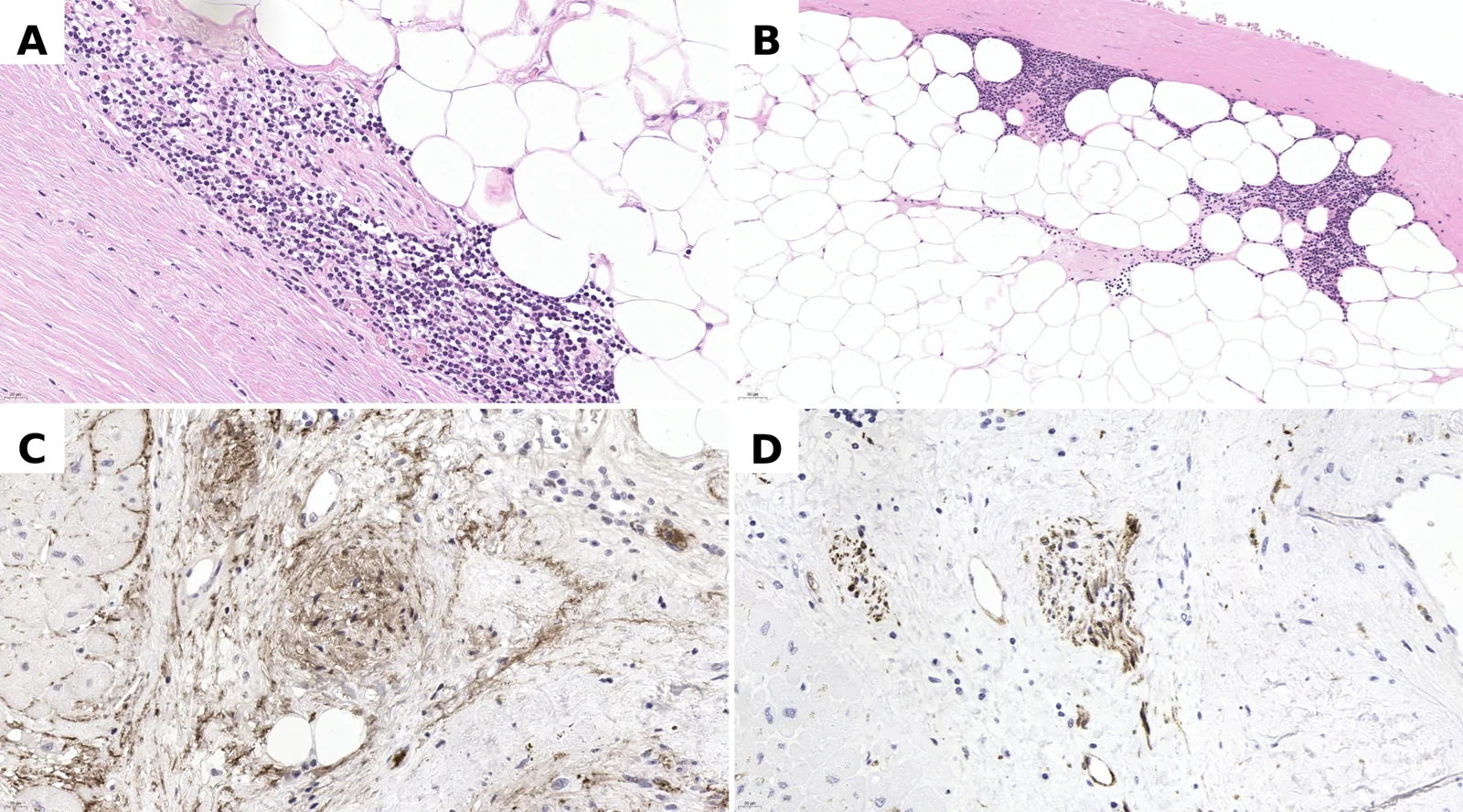
Morphological examination of nerve fibers. (**A**) Dense lymphocytic infiltration at the periphery of a nerve fiber with invasion into the perineurium, hematoxylin and eosin staining, ×400; (**B**) dense lymphoplasmacytic infiltrates in the pericardium and a scarred nerve fibre with isolated perineural cells, haematoxylin and eosin staining, ×200; (**C**) C4d expression within a nerve fiber and in adjacent vessels, where the reaction product is determined both along the fibers and on inflammatory cells, which indirectly indicates the activation of complement in these areas, ×400; (**D**) HLA-DR expression within a nerve fiber and on inflammatory cells at the periphery, ×400.

Against this background, injury to neural fibers was diffuse and pronounced. Histologically, dense lymphocytic and lymphoplasmacytic infiltrates were observed at the periphery of nerve fibers, with inflammatory cell infiltration into the perineurium and presence within the endoneurial space. In some cases, scarred nerve fibers with loss of normal architectural organization and isolated perineural cells were identified, indicating chronic injury and incomplete regeneration. Immunohistochemical analysis demonstrated expression of C4d and C3 within individual nerve fibers and adjacent vessels, as well as HLA-DR expression in neural structures, indicating direct involvement of neural tissue in both humoral and cellular rejection processes (Figure 5).

In addition, innervation of the aorta was evaluated on autopsy material at three levels: the recipient aortic ring, donor aortic ring 1, and donor aortic ring 2. Nerve fibers were identified using a pretrained neural network followed, where necessary, by manual correction of the position and area of individual nerve fibers [16].

Quantitative analysis of aortic innervation at three anatomical levels revealed marked differences reflecting sequential changes characteristic of denervation processes and subsequent remodeling of neural structures within the transplantation complex.

At the level of the recipient aortic ring (above the vascular suture line), the highest number of innervated areas was identified (*n* = 22), predominantly represented by small neural structures. The median area of neural elements was 132.2 μm^2^ (interquartile range 43.7–189.7 μm^2^). Focal clustering of nerve fibers with relatively uniform distribution and small individual size was observed, which may be interpreted as a relatively preserved, near-physiological pattern of innervation in this segment.

In the donor 1 aortic segment (between the two suture lines), a marked reduction in the number of innervated areas was observed (*n* = 7), indicating pronounced denervation in this region. At the same time, the size of the detected neural structures was increased compared with the proximal segment: the median area reached 178.9 μm^2^ (interquartile range 127.1–254.8 μm^2^). This increase in size was not accompanied by a proportional increase in number, reflecting a sparse distribution of nerve fibers in the context of substantial loss of innervation.

**Figure 6:**
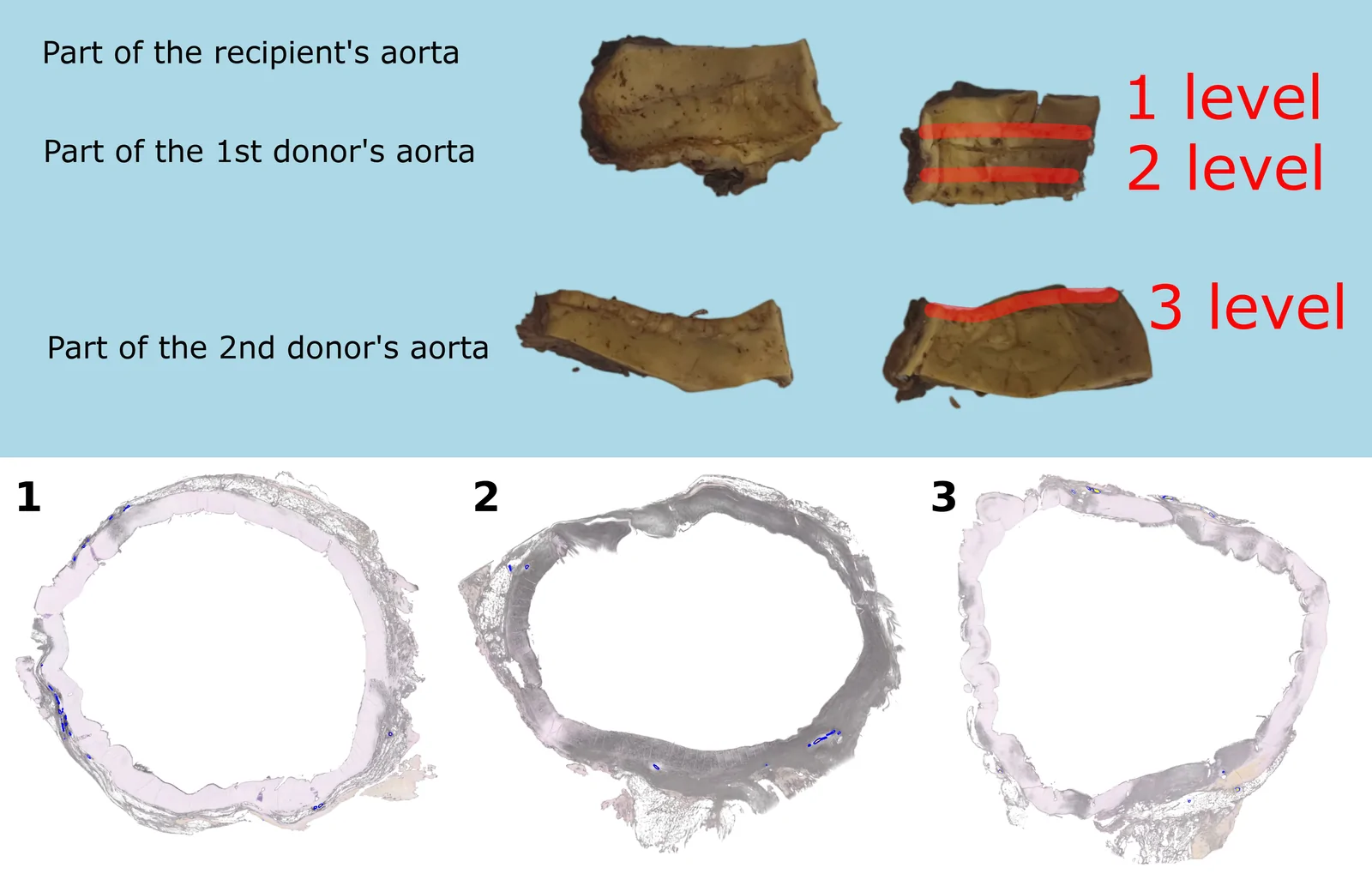
Quantification of the number and density of nerve fibers in the aorta (reconstruction of histological sections) following double heart transplantation. Nerve fibers are indicated in blue; red markings on part of the macroscopic image indicate the levels at which aortic tissue sampling was performed.

At the donor 2 aortic level (at the entry to the left ventricle), signs of denervation persisted; however, the morphology of neural elements differed substantially from the previous segment. The number of innervated areas slightly increased (*n* = 10), although it did not reach the level observed in the recipient aorta, while the formation of larger neural structures was noted, with a median area of 343.2 μm^2^ (interquartile range 172.4–517.1 μm^2^). The emergence of enlarged neural elements against a background of persistently low innervation density may reflect regenerative changes in nerve fibers, potentially associated with the response to acute transection of nerve trunks at the transplantation anastomosis site (Figure 6).

For comparative analysis of innervation density, pixel-by-pixel comparison of local neural fiber density maps was performed between the studied transplant and a control myocardial sample obtained from a 7-year-old boy without cardiovascular disease [17]. This control specimen was selected to provide a spatial reference for the distribution of nerve fibers rather than an age-matched physiological standard. In our institution, which is dedicated to the treatment of cardiovascular diseases, access to myocardial tissue without cardiovascular pathology is extremely limited. Furthermore, large-format histological reconstruction combined with immunohistochemical assessment requires adequately preserved tissue of sufficient size, making suitable control material exceptionally difficult to obtain. Consequently, comparisons were intended to illustrate relative spatial patterns of innervation after normalization rather than to establish absolute age-independent differences in nerve density.

Quantification was performed using an automated algorithm with predefined parameters rather than manual observer-dependent measurements. Digital whole-slide images were analysed at full resolution while preserving the original spatial coordinates of each pixel. Image processing was performed using custom Python 3.12 scripts. Neural fibres were identified based on UCHL-1 immunoreactivity using colour-based segmentation in the HSV colour space, with calibration performed according to the staining intensity of specific neural structures. Analysis was restricted to the myocardial tissue mask to exclude background signal, artefacts, and non-tissue regions.

For each section, a normalized intensity map was generated, followed by application of a weak-signal threshold to remove pixels corresponding to non-specific or insufficient immunoreactivity. Pixels below the predefined intensity threshold (intensity_threshold) were classified as background and excluded from further analysis. Subsequently, a binary neural fiber mask was generated, in which pixels corresponding to UCHL-1-positive nerve fibers were assigned a value of 1 and all other pixels a value of 0.

Threshold selection was based on visual validation of representative regions with high and low UCHL-1 immunoreactivity and was optimized to minimize inclusion of non-specific staining while preserving true neural structures. Since immunohistochemical signal intensity depends on multiple technical factors, including antibody characteristics, dilution, staining platform, detection system, and scanner settings, the optimal threshold is partially assay-specific and cannot be universally transferred between laboratories. Therefore, threshold parameters should be calibrated for each staining protocol before application to independent datasets.

Spatial density maps of neural fibers were constructed using a sliding-window approach. The window size was defined according to absolute tissue area (typically 1 mm^2^) and converted into pixels according to the scanning resolution (µm/pixel). Local innervation density was calculated as the proportion of neural fiber-positive pixels within each window, generating a continuous spatial map of nerve fiber distribution.

Prior to comparison, both maps were normalized to a common value range, ensuring comparability of signal intensities irrespective of staining variability and scanning conditions. For each spatial position, the following parameters were calculated: absolute difference in local density and relative difference normalized to the maximal density value at the given position.

Regions in which the local density of neural fibers was below the threshold value in both maps were classified as background and excluded from interpretation. For the remaining regions, an adaptive difference threshold modified by the algorithm sensitivity parameter was applied. This approach enabled adjustment of classification stringency while accounting for both absolute and relative density changes. Based on these criteria, a difference map was generated identifying three categories of regions: areas of background or extremely low innervation density; areas with comparable neural fiber density; and areas demonstrating marked differences in innervation density between the transplant and the control myocardium.

**Figure 7:**
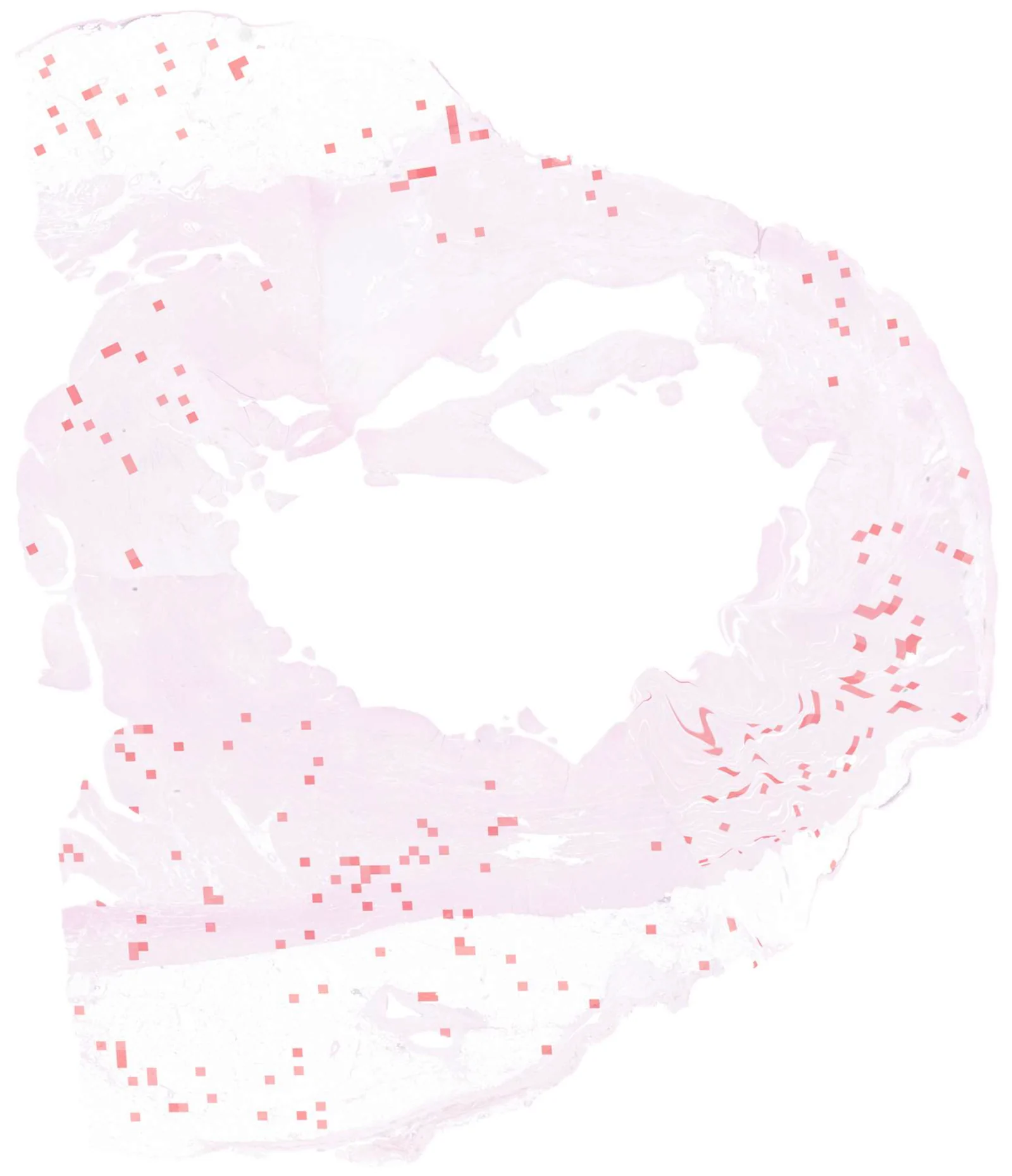
Density map of nerve fibers in the myocardial ring of the heart.

Quantitative assessment of innervation, performed on the basis of UCHL-1-positive nerve fiber density maps, demonstrated a pronounced and heterogeneous reduction in innervation density within the reconstructed cardiac ring (Figure 7). Importantly, large areas of denervation were identified not only in regions of transmural infarction and severe myocardial fibrosis, but also in relatively morphologically preserved myocardial areas. This finding indicates a systemic pattern of neural injury not limited to secondary ischemic damage. In addition, a widespread reduction in nerve fiber density was observed along the vascular periphery, particularly in regions of marked perivascular fibrosis and inflammatory infiltration, as well as an almost complete absence of innervation within the pericardial adipose tissue across most examined areas.

**Figure 8:**
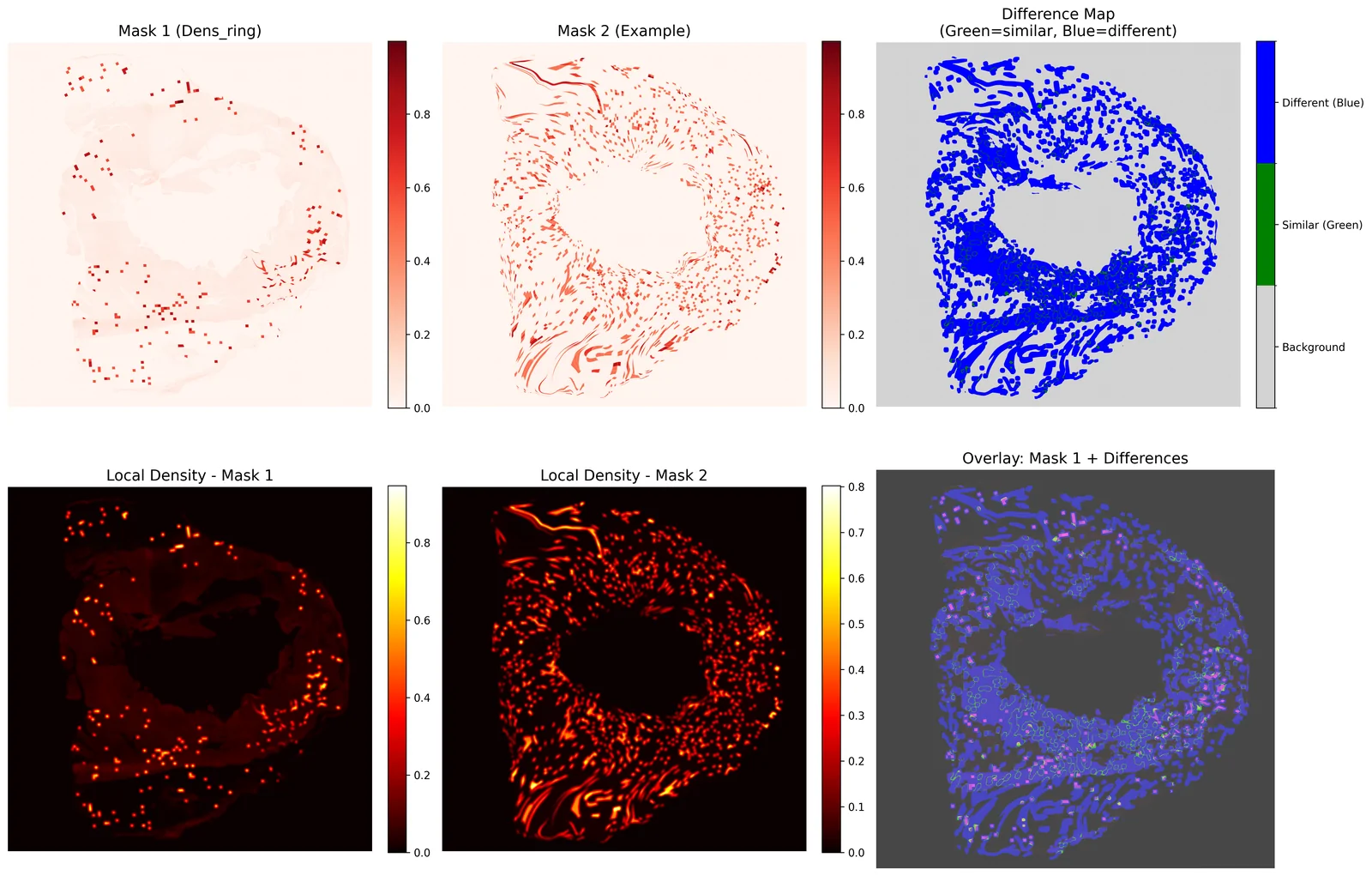
Quantitative visualization of cardiac denervation in chronic allograft rejection: blue indicates areas of marked reduction in innervation density; green indicates areas with preserved density.

Compared with a control myocardial sample obtained from a 7-year-old boy without cardiovascular pathology, a marked expansion of areas with critically low nerve fiber density was observed. Pixel-by-pixel comparison of local density maps demonstrated that the majority of the myocardial ring area of the transplant corresponded to regions of pronounced denervation, visualized as blue areas on the difference map (Figure 8). Regions of relatively preserved innervation density (green areas) were fragmentary in nature and constituted a smaller proportion of the total area, predominantly located at the periphery of focal scars and in proximity to elements of the cardiac conduction system.

## 3. Discussion

Long-term cardiac allograft pathology has traditionally been interpreted primarily through the framework of cardiac allograft vasculopathy (CAV). However, our morphological and quantitative findings demonstrate that injury to the intracardiac nervous system represents an independent and widespread component of chronic graft dysfunction that has previously been under-recognized.

It should be noted that heart transplantation itself is inevitably accompanied by complete surgical transection of sympathetic and parasympathetic nerve fibers, resulting in Wallerian degeneration of axons and primary myocardial denervation. Nevertheless, our results demonstrate that chronic rejection is associated with a secondary denervation caused by chronic graft injury that extends beyond the initial surgical denervation.

Morphological examination of nerve fibers (Figure 5) revealed marked lymphocytic and lymphoplasmacytic infiltration within both the perineurium and endoneurium, accompanied by scarring of nerve trunks. The expression of CD3, C4d and HLA-DR within nerve fibers indicates the concurrent involvement of humoral and cellular immune mechanisms. Quantitative analysis of innervation density (Figure 7 and Figure 8) demonstrated extensive areas of profound loss of nerve fibers not only in regions of fibrosis and prior myocardial infarction, but also outside these areas, indicating a generalized neuropathic process. In addition, a widespread reduction in perivascular innervation and an almost complete absence of nerve fibers within pericardial adipose tissue were observed across most examined regions.

Cell-mediated immunity appears to play a central role in injury to neural structures. Experimental models of peripheral nerve allotransplantation have shown that rejection is initiated by an innate immune response involving activation of macrophages and dendritic cells, which in turn recruit T cells that release pro-inflammatory cytokines, chemokines, and reactive oxygen species [18]. Macrophages and dendritic cells amplify the T-cell response through both direct and indirect allo-recognition pathways [19]. The observed infiltration of CD3+ T cells and CD68+ macrophages in perivascular and perineural regions supports the relevance of these mechanisms to intracardiac nerves.

Recent studies further highlight the complexity of immune regulation during cardiac transplantation. T-cell activity is not only determined by activation pathways but also by functional exhaustion and senescence states. Chen et al. [20] demonstrated that induction of CD4+ T-cell senescence can attenuate alloimmune responses and promote graft tolerance, suggesting that the balance between effector activation and immune regulation may influence the extent of chronic graft injury. Conversely, persistent activation of inflammatory immune pathways may sustain neural damage despite conventional immunosuppression.

Mechanisms of peripheral nerve injury described in sciatic nerve trauma are largely applicable to intracardiac innervation. Upon injury, Schwann cells activate the production of TNF-α, IL-1β, and CCL2, promoting macrophage recruitment. Sustained macrophage infiltration is maintained via multiple parallel signaling pathways (CCL2, Toll-like receptors, ICAM-1, prostaglandins), rendering selective blockade of individual cascades ineffective. Macrophages contribute to demyelination and clearance of myelin debris via complement component C3 and its receptors, as well as to Wallerian degeneration through IL-1β, TNF-α, MCP-1, collagen VI, Nrf2, phospholipase A2, and matrix metalloproteinases MMP-2 and MMP-9 [21].

Humoral immunity and barrier mechanisms modulate the extent of neural injury. Peripheral nerves are partially protected by the blood–nerve barrier, formed by the epineurium and tight endothelial junctions, which limits antibody diffusion [22]. Experimental data indicate that immune infiltration in nerve allografts occurs predominantly from the graft ends rather than along its entire length, reflecting the barrier function of the blood–nerve interface [23]. Moreover, low MHC expression in neural tissue [24] and partial immune privilege of neurons and myelinating Schwann cells [25,26] may account for the relative resistance of nerves; however, these protective mechanisms appear insufficient in the setting of chronic rejection.

Ischemic injury likely represents an additional factor accelerating neural degeneration. Under physiological conditions, cardiomyocytes maintain sympathetic innervation through secretion of nerve growth factor (NGF) at neuro–cardiac junctions. Myocardial injury disrupts this interaction, leading to NGF deprivation and degeneration of sympathetic fibers [27]. Following myocardial infarction, neural remodeling demonstrates an initial phase of nerve loss followed by inflammation-associated hyperinnervation in peri-infarct regions, whereas scar tissue remains resistant to reinnervation due to inhibitory extracellular matrix components such as chondroitin sulphate proteoglycans [28]. Similar processes may occur in cardiac allografts affected by CAV, chronic ischemia, and ischemia–reperfusion injury, contributing to the irreversible reduction in neural density observed in our study.

Inflammatory signaling pathways associated with oxidative stress and inflammasome activation may provide a molecular link between ischemia, immune activation, and neural degeneration. Activation of the NLRP3 inflammasome in Schwann cells promotes pyroptotic cell death with release of IL-1β and IL-18, thereby amplifying local neuroinflammation [29,30]. Under ischemic inflammatory conditions, activation of the CXCL12/CXCR4/NF-κB pathway may further enhance NLRP3-dependent pyroptosis [31,32], contributing to demyelination and axonal loss [33]. The involvement of these pathways is supported by broader cardiovascular literature demonstrating the importance of inflammasome-mediated inflammation.

Wang et al. [34] further emphasized the contribution of NLRP3-dependent macrophage activation and proinflammatory responses in chronic vascular inflammation. In addition, Bao et al. [35] demonstrated that regulation of inflammatory macrophage responses through the TNK1/Tyk2/STAT1 axis contributes to atherosclerotic pathology, providing a potential mechanistic link between vascular inflammation, immune activation, and chronic graft injury.

Finally, pharmacological factors may further aggravate neural damage. Calcineurin inhibitor (CNI)-associated neurotoxicity has been described in transplant recipients, with neuropathy reported more frequently in patients receiving CNI-based regimens compared with alternative immunosuppressive approaches [36]. Proposed mechanisms include endothelial dysfunction, vasoconstriction, impaired microcirculation, and energetic deficiency within neural tissue. In cardiac allografts, these effects may act synergistically with CAV-associated ischemia and chronic immune-mediated inflammation, accelerating degeneration of intracardiac nerves and limiting the potential for functional reinnervation.

Our findings are broadly consistent with the current concept that cardiac reinnervation after heart transplantation is incomplete, spatially heterogeneous, and varies substantially between patients. Comprehensive physiological and imaging studies have demonstrated that although sympathetic reinnervation may gradually develop over time, complete restoration of cardiac autonomic innervation rarely occurs even many years after transplantation, with denervated myocardial regions persisting in most recipients [14]. A recent large cohort study by Weiner et al. reported evidence of sympathetic reinnervation in approximately 70% of recipients more than 18 months after transplantation, with reinnervation occurring predominantly during the first 18 months and being associated with significantly improved long-term survival [37]. Importantly, these studies evaluate functional sympathetic activity rather than the structural integrity of nerve fibers. In contrast, our histopathological analysis directly demonstrates extensive structural loss of intracardiac nerves, accompanied by inflammatory infiltration, perineural fibrosis, and marked reduction in nerve fiber density. Furthermore, unlike physiological studies that generally assess global cardiac autonomic function, our quantitative whole-section mapping revealed pronounced regional heterogeneity, with extensive denervation not only within the infarcted and fibrotic myocardium but also in morphologically preserved areas, suggesting that chronic graft injury affects neural structures beyond regions of overt ischemic remodeling.

These observations are also in agreement with molecular imaging studies using PET. Hybrid PET/MRI investigations have demonstrated that sympathetic reinnervation is patchy and limited to discrete myocardial segments rather than occurring uniformly throughout the transplanted heart [5]. Beitzke et al. identified evidence of sympathetic reinnervation in only a minority of myocardial segments and demonstrated substantial regional variability in tracer uptake, indicating heterogeneous recovery of sympathetic fibers.

Thus, the present findings suggest that injury to intracardiac innervation may represent an under-recognized component of chronic cardiac allograft remodeling. At present, comprehensive pathological assessment of cardiac innervation is primarily applicable to explanted hearts and post-mortem examinations, where it may improve understanding of the mechanisms underlying graft failure. In addition, selected morphological features of neural injury could potentially be incorporated into the evaluation of routine endomyocardial biopsy specimens obtained for surveillance of cellular and antibody-mediated rejection, although the feasibility and diagnostic value of this approach remain to be established.

From a translational perspective, the observed pathological changes also provide a rationale for future studies investigating whether non-invasive assessment of cardiac innervation using established functional imaging techniques, such as PET or SPECT, could serve as a surrogate marker of advanced chronic graft injury or cardiac allograft vasculopathy. Should such associations be confirmed, imaging of cardiac innervation might contribute to risk stratification and monitoring of transplant recipients. However, these potential clinical applications remain speculative and require validation in larger studies integrating imaging, pathology, and clinical outcomes.

## 4. Study Limitations

Several limitations of this study should be acknowledged. First, the analysis was based on a single explanted cardiac allograft, which limits generalisability and prevents assessment of the prevalence and variability of intracardiac neural injury in the transplant population. However, the extensive spatial reconstruction and quantitative mapping provide detailed insight into the distribution of neural loss associated with advanced chronic rejection.

Second, the reference tissue used for comparison was obtained from a pediatric heart and therefore does not represent an age-matched normal adult control. Differences in age, developmental stage, and individual variability in cardiac neural architecture should be considered; accordingly, these comparisons should be interpreted as a relative spatial framework rather than an absolute physiological baseline.

Third, this study assessed structural neural alterations using histological and immunohistochemical methods, but functional consequences of denervation, including autonomic regulation, electrophysiological changes, and neurocardiac interactions, were not evaluated. In addition, the cross-sectional design does not allow causal relationships to be established between immune activation, ischemic injury, and neural degeneration.

Finally, although immunohistochemical findings suggest involvement of inflammatory and immune-mediated mechanisms, complementary molecular analyses, such as transcriptomic, proteomic, or targeted pathway assessment, were not performed. Therefore, the proposed mechanisms, including inflammasome activation and pyroptosis, require further validation in larger studies integrating morphological, molecular, and functional approaches.

## 5. Conclusions

This case demonstrates that long-term cardiac allograft pathology may involve not only cardiac allograft vasculopathy but also extensive structural injury to the intracardiac nervous system. Morphological and quantitative analyses revealed widespread reduction in nerve fiber density affecting fibrotic and ischaemic regions as well as a relatively preserved myocardium, with additional involvement of perivascular regions and pericardial adipose tissue.

The presence of perineural and endoneurial inflammatory infiltrates together with immunohistochemical evidence of complement deposition and immune activation suggests that intracardiac nerve fibers may be directly involved in the pathological processes accompanying chronic cardiac allograft rejection. The quantitative mapping approach further illustrates the potential value of spatial assessment of cardiac innervation in characterizing neural remodeling in transplanted hearts.

Although these observations are derived from a single explanted allograft and therefore cannot establish general mechanisms of chronic graft dysfunction, they generate the hypothesis that progressive injury to the intracardiac nervous system may represent an under-recognized component of chronic allograft remodeling. Confirmation of this hypothesis will require larger studies integrating pathological, imaging, and clinical data to determine the prevalence, mechanisms, and potential clinical significance of neural injury after heart transplantation.

## Data Availability

If you need clarifications, or need additional information, you can write to the email: doctormakarovia@gmail.com.

## References

[1] Uberfuhr P., Ziegler S., Schwaiblmair M., Reichart B., Schwaiger M. (2000). Incomplete sympathic reinnervation of the orthotopically transplanted human heart: Observation up to 13 years after heart transplantation. Eur. J. Cardio-Thorac. Surg..

[2] Gallego-Page J.C., Segovia J., Alonso-Pulpón L., Alonso-Rodríguez M., Salas C., Ortíz-Berrocal J. (2004). Re-innervation after heart transplantation: A multidisciplinary study. J. Heart Lung Transplant..

[3] Bengel F.M., Ueberfuhr P., Schiepel N., Nekolla S.G., Reichart B., Schwaiger M. (2001). Effect of sympathetic reinnervation on cardiac performance after heart transplantation. N. Engl. J. Med..

[4] Schwaiblmair M., von Scheidt W., Uberfuhr P., Ziegler S., Schwaiger M., Reichart B., Vogelmeier C. (1999). Functional significance of cardiac reinnervation in heart transplant recipients. J. Heart Lung Transplant..

[5] Beitzke D., Wielandner A., Wollenweber T., Vraka C., Pichler V., Uyanik-Uenal K., Zuckermann A., Greiser A., Hacker M., Loewe C. (2019). Assessment of sympathetic reinnervation after cardiac transplantation using hybrid cardiac PET/MRI: A pilot study. J. Magn. Reson. Imaging.

[6] Bengel F.M., Ueberfuhr P., Schiepel N., Nekolla S.G., Reichart B., Schwaiger M. (2001). Myocardial efficiency and sympathetic reinnervation after orthotopic heart transplantation: A noninvasive study with positron emission tomography. Circulation.

[7] Watanabe K., Fukuchi K., Echigo S. (2004). Early sympathetic reinnervation demonstrated by iodine-123 metaiodobenzylguanidine imaging in a child after cardiac transplantation. Pediatr. Cardiol..

[8] Kim D.T., Luthringer D.J., Lai A.C., Suh G., Czer L., Chen L.S., Chen P.-S., Fishbein M.C. (2004). Sympathetic nerve sprouting after orthotopic heart transplantation. J. Heart Lung Transplant..

[9] Ludwig J., Friedgen B., Herrmann G., Zahorsky R., Inselmann G., Simon R., Graefe K.H., Nellessen U. (1994). Evidence for partial sympathetic cardiac reinnervation following cardiac transplantation. Eur. J. Cardio Thorac. Surg..

[10] Bengel F.M., Ueberfuhr P., Schäfer D., Nekolla S.G., Reichart B., Schwaiger M. (2006). Effect of diabetes mellitus on sympathetic neuronal regeneration studied in the model of transplant reinnervation. J. Nucl. Med..

[11] Awad M., Czer L.S., Hou M., Golshani S.S., Goltche M., De Robertis M., Kittleson M., Patel J., Azarbal B., Kransdorf E. (2016). Early Denervation and Later Reinnervation of the Heart Following Cardiac Transplantation: A Review. J. Am. Heart Assoc..

[12] Toba M., Ishida Y., Fukuchi K., Shimotsu Y., Takamiya M., Komamura K., Nakatani T., Ohuchi H., Ono Y., Kamiya T. (1998). Sympathetic reinnervation demonstrated on serial iodine-123-metaiodobenzylguanidine SPECT images after cardiac transplantation. J. Nucl. Med..

[13] Vallejo Venegas E. (2000). Nuclear cardiology and its application in the study of the patient undergoing cardiac transplant. Arch. Inst. Cardiol. Mex..

[14] Asleh R., Alnsasra H., Villavicencio M.A., Daly R.C., Kushwaha S.S. (2023). Cardiac Transplantation: Physiology and Natural History of the Transplanted Heart. Compr. Physiol..

[15] Grupper A., Gewirtz H., Kushwaha S. (2018). Reinnervation post-heart transplantation. Eur. Heart J..

[16] Makarov I., Koshevaya E., Pechenina A., Boyko G., Starshinova A., Kudlay D., Makarova T., Mitrofanova L. (2025). A Comparative Analysis of SegFormer, FabE-Net and VGG-UNet Models for the Segmentation of Neural Structures on Histological Sections. Diagnostics.

[17] Makarov I., Solovyova O., Starshinova A., Kudlay D., Mitrofanova L. (2026). SKUF Protocol: Slice, Keep, Unwrap, Fuse-A Pilot Multimodal Approach to Cardiac Innervation Mapping. Diagnostics.

[18] Gunasekaran M., Vachharajani N., Gaut J.P., Maw T.T., Delos Santos R., Shenoy S., Chapman W.C., Wellen J., Mohanakumar T. (2017). Development of immune response to tissue-restricted self-antigens in simultaneous kidney-pancreas transplant recipients with acute rejection. Clin. Transplant..

[19] Marino J., Paster J., Benichou G. (2016). Allorecognition by T Lymphocytes and Allograft Rejection. Front. Immunol..

[20] Chen Y., Yim W.Y., Li C., Wang Z., Hou J., Peng Y., Liu F., Liu Z., Dong N., Wang Y. (2026). T cell senescence induced by DNMT1 deletion mediates cardiac transplant tolerance via altered CDKN1A methylation. Mol. Ther..

[21] Xia Y., Cai M., Zhou Y., Yao Y., Jiang M., Gu D., Yao D. (2025). Immune Cell Biology in Peripheral Nervous System Injury. Neurorehabilit. Neural Repair.

[22] Maiuolo J., Gliozzi M., Musolino V., Carresi C., Nucera S., Macrì R., Scicchitano M., Bosco F., Scarano F., Ruga S. (2019). The Role of Endothelial Dysfunction in Peripheral Blood Nerve Barrier: Molecular Mechanisms and Pathophysiological Implications. Int. J. Mol. Sci..

[23] Santos Roballo K.C., Dhungana S., Jiang Z., Oakey J., Bushman J.S. (2019). Localized delivery of immunosuppressive regulatory T cells to peripheral nerve allografts promotes regeneration of branched segmental defects. Biomaterials.

[24] Trumble T.E., Gunlikson R., Parvin D. (1994). Systemic immune response to peripheral nerve transplants across major histocompatibility class-I and class-II barriers. J. Orthop. Res..

[25] Nardo G., Trolese M.C., Bendotti C. (2016). Major Histocompatibility Complex I Expression by Motor Neurons and Its Implication in Amyotrophic Lateral Sclerosis. Front. Neurol..

[26] Lisak R.P., Bealmear B., Benjamins J.A. (2016). Schwann cell differentiation inhibits interferon-gamma induction of expression of major histocompatibility complex class II and intercellular adhesion molecule-1. J. Neuroimmunol..

[27] Dokshokova L., Franzoso M., Di Bona A., Moro N., Sanchez Alonso J.L., Prando V., Sandre M., Basso C., Faggian G., Abriel H. (2022). Nerve growth factor transfer from cardiomyocytes to innervating sympathetic neurons activates TrkA receptors at the neuro-cardiac junction. J. Physiol..

[28] Yu T., Pei B., Zhao D. (2025). Sympathetic Biomarker Dynamics Post-Myocardial Infarction: TH, PGP9.5, and SYN Expression Discordance in Murine Hearts. Int. J. Mol. Sci..

[29] Cheng Y.C., Chu L.W., Chen J.Y., Hsieh S.L., Chang Y.C., Dai Z.K., Wu B.N. (2020). Loganin Attenuates High Glucose-Induced Schwann Cells Pyroptosis by Inhibiting ROS Generation and NLRP3 Inflammasome Activation. Cells.

[30] Lamkanfi M., Dixit V.M. (2014). Mechanisms and functions of inflammasomes. Cell.

[31] Hou J., Wang C., Ma D., Chen Y., Jin H., An Y., Jia J., Huang L., Zhao H. (2021). The cardioprotective and anxiolytic effects of Chaihujialonggumuli granule on rats with anxiety after acute myocardial infarction is partly mediated by suppression of CXCR4/NF-κB/GSDMD pathway. Biomed. Pharmacother..

[32] Starshinova A., Fedotov P., Bulgun M., Kudryavtsev I., Rubinstein A., Aquino A.D., Kudlay D., Shlyakhto E. (2026). Cardiac sarcoidosis: From clinical manifestations to heart transplantation. Front. Med..

[33] Li W., Liang J., Li S., Jiang S., Song M., Xu S., Wang L., Meng H., Zhai D., Tang L. (2023). The CXCL12-CXCR4-NLRP3 axis promotes Schwann cell pyroptosis and sciatic nerve demyelination in rats. Clin. Exp. Immunol..

[34] Wang M., Cheng J., Feng F., Chen L. (2026). NLRP3 Inflammasome: A Potential Therapeutic Target for Atherosclerosis. Health Metab..

[35] Bao M.H., Lv Q.L., Li H.G., Zhang Y.W., Xu B.F., He B.S. (2020). A Novel Putative Role of TNK1 in Atherosclerotic Inflammation Implicating the Tyk2/STAT1 Pathway. Mediat. Inflamm..

[36] Veroux P., Veroux M., Puliatti C., Morale W., Cappello D., Valvo M., Macarone M. (2002). Tacrolimus-induced neurotoxicity in kidney transplant recipients. Transplant. Proc..

[37] Weiner O.J.F., Das M., Clayton R.H., McComb J.M., Murray A., Parry G., Lord S.W. (2025). Sympathetic reinnervation in cardiac transplant recipients: Prevalence, time course, and association with long-term survival. J. Heart Lung Transplant..

